# The Active Glucuronide Metabolite of the Brain Protectant IMM-H004 with Poor Blood–Brain Barrier Permeability Demonstrates a High Partition in the Rat Brain via Multiple Mechanisms

**DOI:** 10.3390/pharmaceutics16030330

**Published:** 2024-02-27

**Authors:** Jianwei Jiang, Lijun Luo, Ziqian Zhang, Xiao Liu, Naihong Chen, Yan Li, Li Sheng

**Affiliations:** 1 State Key Laboratory of Bioactive Substances and Functions of Natural Medicines, Institute of Materia Medica, Chinese Academy of Medical Sciences and Peking Union Medical College, Beijing 100050, China; jwjiang98@zju.edu.cn (J.J.); luolijun@imm.ac.cn (L.L.);; 2 Department of Drug Metabolism, Institute of Materia Medica, Chinese Academy of Medical Sciences and Peking Union Medical College, Beijing 100050, China; 3 Beijing Key Laboratory of Non-Clinical Drug Metabolism and PK/PD Study, Institute of Materia Medica, Chinese Academy of Medical Sciences and Peking Union Medical College, Beijing 100050, China; 4 Department of Pharmacology, Institute of Materia Medica, Chinese Academy of Medical Sciences and Peking Union Medical College, Beijing 100050, China

**Keywords:** blood–brain barrier, glucuronide, conjugate metabolite, uridine diphosphate-glucuronosyltransferase, organic anion-transporting polypeptide

## Abstract

Background: Glucuronidation is an essential metabolic pathway for a variety of drugs. IMM-H004 is a novel neuroprotective agent against ischemic stroke, and its glucuronide metabolite IMM-H004G exhibits similar pharmacological activity. Despite possessing a higher molecular weight and polarity, brain exposure of IMM-H004G is much higher than that of IMM-H004. This study aimed to investigate the brain metabolism and transport mechanisms of IMM-H004 and IMM-H004G. Methods: First, the possibility of IMM-H004 glucuronidation in the brain was evaluated in several human brain cell lines and rat homogenate. Subsequently, the blood–brain barrier carrier-mediated transport mechanism of IMM-H004 and IMM-H004G was studied using overexpression cell models. In addition, intracerebroventricular injection, in situ brain perfusion model, and microdialysis/microinjection techniques were performed to study the distribution profiles of IMM-H004 and IMM-H004G. Results: IMM-H004 could be metabolized to IMM-H004G in both rat brain and HEB cells mediated by UGT1A7. However, IMM-H004G could not be hydrolyzed back into IMM-H004. Furthermore, the entry and efflux of IMM-H004 in the brain were mediated by the pyrilamine-sensitive H^+^/OC antiporter and P-gp, respectively, while the transport of IMM-H004G from the blood to the brain was facilitated by OATP1A2 and OATP2B1. Ultimately, stronger concentration gradients and OATP-mediated uptake played a critical role in promoting greater brain exposure of IMM-H004G. Conclusions: The active glucuronide metabolite of the brain protectant IMM-H004 with poor blood–brain barrier permeability demonstrates a high partition in the rat brain via multiple mechanisms, and our findings deepen the understanding of the mechanisms underlying the blood–brain barrier metabolism and transport of active glucuronide conjugates.

## 1. Introduction

According to the World Stroke Organization, the incidence of stroke currently ranks second among the leading causes of death and third in terms of combined mortality and disability worldwide in 2022 [1]. Thrombolytic tissue plasminogen activator (tPA) has been the only agent approved by the Food and Drug Administration (FDA) for the treatment of ischemic stroke. However, the narrow time window for thrombolytic treatment (4.5 h) and the risk of hemorrhagic transformation limit the number of stroke patients who can benefit from tPA [2]. Therefore, there is an urgent need for neuroprotective agents that have extended treatment time windows and fewer side effects.

IMM-H004, a novel 3-piperazinyl coumarin derivative, is currently under development for the treatment of cerebral ischemia. Previous studies have shown that administering IMM-H004 6 h after cerebral ischemia can prevent brain damage by activating the anti-inflammatory pathway of CKLF1/CCR4 [3,4]. IMM-H004 has also been found to protect against global cerebral ischemia in rats by inhibiting apoptosis and maintaining the integrity of synaptic structures [5]. Additionally, it significantly reduces cerebral ischemia–reperfusion injury and subsequent inflammation in spontaneously hypertensive rats [6]. These findings suggest that IMM-H004 holds promise as a treatment for cerebral ischemia.

**Figure 1:**
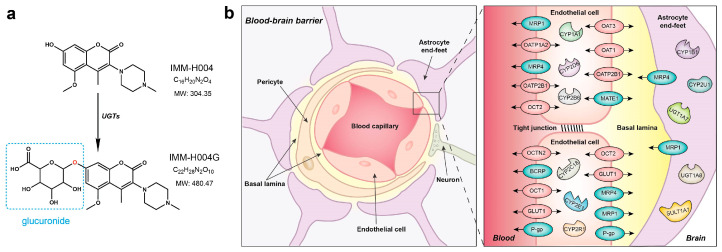
Structures of IMM-H004/IMM-H004G and the blood–brain barrier: (**a**) partial chemical information of IMM-H004 and IMM-H004G; (**b**) schematic diagram of the structure of the blood–brain barrier. Abbreviations: BCRP, breast cancer resistance protein; CYP, cytochrome P450; GLUT, glucose transporter; MATE, multidrug and toxin extrusion transporter; MRP, multidrug resistance protein; OAT, organic anion transporter; OATP, organic anion-transporting polypeptide; OCT, organic cation transporter; P-gp, P-glycoprotein; SULT, sulfotransferase; UGT, uridine diphosphate glucuronosyltransferase.

Despite its effectiveness, IMM-H004 has a very short elimination half-life (<1 h) in rat plasma and brain tissues after intravenous dosing [7]. Subsequent studies have identified four metabolites of IMM-H004 in rats, namely two demethylated metabolites, a glucuronide conjugate (IMM-H004G), and a sulfated conjugate. Among these metabolites, IMM-H004G is found to be the major metabolite in both rats and cultured human hepatocytes (Figure 1a). Multiple UDP glucuronosyltransferases (UGTs) are involved in the formation of IMM-H004G, with the highest contribution of UGT1A7, followed by UGT1A9, 1A8, and 1A1, whereas UGT1A3, 1A10, 2B15 and 1A6 exhibited less activity toward IMM-H004G [7]. Interestingly, brain exposure of IMM-H004G in rats was found to be six times higher than that of the parent drug [8]. Furthermore, pharmacological studies have shown that IMM-H004G exhibits neuroprotective activity similar to that of the parent drug in both oxygen–glucose-deprived PC12 cells and rats with transient middle cerebral artery occlusion–reperfusion (MCAO/R) injury. Hence, after administering IMM-H004, the active glucuronide metabolite IMM-H004G may prolong the duration of drug action and contribute to the efficacy of anticerebral ischemia treatment.

Generally, molecules with high lipophilicity and small molecular size can easily diffuse across the blood–brain barrier (BBB) following the concentration gradient [9]. Compared to IMM-H004, IMM-H004G is a highly polar and hydrophilic compound with a large molecular weight. However, brain exposure of IMM-H004G is significantly higher than that of IMM-H004. Due to its physicochemical properties, IMM-H004G is not favored for passive diffusion across the BBB, suggesting that it may depend on a transport system to reach high concentrations in the brain (Figure 1b). Additionally, it is widely recognized that during the transportation across the BBB, drugs have the potential to undergo metabolic transformations facilitated by drug-metabolizing enzymes that are present within the BBB [10]. Therefore, does the high brain exposure of IMM-H004G result from the in situ metabolism of IMM-H004, or is it transported into the brain through the BBB?

In this study, the impact of drug-metabolizing enzymes and transporters on the brain distribution of IMM-H004 and IMM-H004G was evaluated. This work strengthens our understanding of the metabolic and transport mechanisms of active glucuronide metabolites in the brain, which will be fundamental to the investigation of the toxicity and efficacy of novel neuroprotective agents while also promoting further research into active glucuronide compounds.

## 2. Materials and Methods

### 2.1. Chemicals and Materials

IMM-H004 and IMM-H004 citrates (purity > 99%) were synthesized by the Laboratory of Chemical Synthesis, and IMM-H004G (purity > 99%) was provided by the Laboratory of Biosynthesis of Natural Products, Institute of Materia Medica, Chinese Academy of Medical Science, as described previously [7,8]. Propranolol hydrochloride (internal standard, IS), digoxin, mitoxantrone, methotrexate, tetraethylammonium chloride (TEA), and metformin hydrochloride were purchased from Sigma-Aldrich (St. Louis, MO, USA). Diphenhydramine (DPH), cimetidine, quinidine, verapamil hydrochloride, L-carnitine, and benzbromarone were purchased from J&K Scientific, Ltd. (Beijing, China). Atenolol, pyrimethamine, rosuvastatin calcium (RSV), estradiol 17-(β-D-glucuronide) (E17βG), rifampicin (RFP), PSC833, MK571, and Ko143 were purchased from MedChemExpress (Monmouth Junction, NJ, USA). Estrone 3-sulfate sodium (E3S) was obtained from Krre Inc. (Beijing, China). Rifamycin SV was purchased from China National Institutes for Food and Drug Control. Type I collagen from rat tail was purchased from Thermo Fisher Scientific (Waltham, MA, USA). The 0.25% trypsin–EDTA solution was purchased from Gibco (Carlsbad, CA, USA). The transwell insert (0.4 μm) was obtained from Millipore (Billerica, MA, USA). The SH-SY5Y cell line was obtained from the Cell Resource Center, Peking Union Medical College (Beijing, China). The normal human astrocyte cell line HEB was purchased from the Experimental Animal Center of Sun Yat-Sen University of Medical Sciences (Guangzhou, China). Immortalized human brain microvascular endothelial cells hCMEC/D3 and related culture media were purchased from Millipore (Billerica, MA, USA). Madin–Darby canine kidney Ⅱ (MDCKⅡ) cells were purchased from the American Type Culture Collection (Manassas, VA, USA). Wild-type Chinese hamster ovary (CHO) cells were a gift from Prof. Naihong Chen (Institute of Materia Medica, Chinese Academy of Medical Sciences and Peking Union Medical College, Beijing, China). The passage number of the cell lines was between 5 and 20.

### 2.2. Animals

All animal studies were approved by the Animal Care and Welfare Committee of Peking Union Medical College (approval code: 00007839) and strictly adhered to the guidelines for the use and care of laboratory animals issued by the Institute Animal Care and Welfare Committee. Male Sprague Dawley rats (260–280 g) were purchased from Vital River Experimental Animal Co., Ltd. (Beijing, China). The animals were acclimated for one week prior to the treatment and provided with free access to water and food.

### 2.3. Metabolism in the Brain

#### 2.3.1. In Vitro Metabolism in Human Brain Cells and Rat Brain Homogenate

SH-SY5Y, HEB, and hCMEC/D3 cells were utilized to investigate the in vitro metabolism of IMM-H004 and IMM-H004G in the brain. SH-SY5Y and HEB cells were cultured routinely in Dulbecco’s modified Eagle’s medium (DMEM) containing 10% fetal bovine serum and a 100 U/mL penicillin–100 μg/mL streptomycin solution. hCMEC/D3 cells were seeded on collagen type I coated 24-well plates and cultured in EndoGRO™-MV complete medium following the manufacturer’s guidelines. The cells were incubated at 37 °C, with 5% CO_2_, and were maintained in a humidified environment. Once the confluence reached 70–80%, the cells were digested using a 0.25% trypsin–EDTA solution, centrifuged at 200× *g* for 5 min, and then resuspended in a fresh culture medium. For each experiment, after seeding and 24 h incubation, the cells were washed twice with a prewarmed culture medium, and then IMM-H004 or IMM-H004G (1–100 μmol/L) dissolved in the culture medium was added. Incubation was carried out at 37 °C for 5 h or 24 h. After incubation, the supernatants were collected. Subsequently, the cells were washed with cold HBSS three times and lysed in a RIPA lysis buffer (Beyotime Inc., Shanghai, China). The concentrations of IMM-H004 and IMM-H004G were determined by liquid chromatography with tandem mass spectrometry (LC-MS/MS).

Rat brains were homogenized in chilled saline (1:3, *w*/*v*) on ice to obtain the brain homogenate. The incubation mixtures composed of the brain homogenate (2 mg protein/mL), alamethicin (50 μg/mg protein), IMM-H004 (10 μmol/L), and UDPGA (5 mmol/L) in a final volume of 0.2 mL of Tris-HCl buffer (50 mmol/L, pH 7.4) containing 5 mmol/L of MgCl_2_. Before initiating the reaction, the brain homogenate was preincubated with alamethicin on ice for 15 min. Subsequently, IMM-H004 and UDPGA were added to initiate the reaction. After incubating at 37 °C for 60 min, the reaction was quenched by adding two volumes of acetonitrile containing IS (0.1 μg/mL). On the other hand, IMM-H004G (1 μmol/L) was incubated with the brain homogenate (2 mg protein/mL) for up to 4 h. The concentrations of IMM-H004 and IMM-H004G were determined by LC-MS/MS.

#### 2.3.2. Quantification of UGT Expression in SH-SY5Y and HEB Cells by qRT-PCR

The expression levels of UGT1A1, UGT1A3, UGT1A6, UGT1A7, UGT1A8, UGT1A9, UGT1A10, and UGT2B15 in SH-SY5Y and HEB cells were compared using qRT-PCR. Total RNA was isolated using the RNeasy micro kit (Qiagen, Hilden, Germany) following the manufacturer’s instructions. The concentrations and purity of the RNA samples were accessed using a NanoPhotometer (Implen Gmbh, Munich, Germany). cDNA synthesis was performed using High-Capacity cDNA Reverse Transcription Kits (Applied Biosystems, Wakefield, RI, USA). For each reaction, 1 μg of cDNA was combined with a universal master mix, sense and antisense primers (0.4 μmol/L each), and oligonucleotide probes (0.2 μmol/L). Primer and probe for UGTs and GAPDH were purchased from Thermo Fisher Scientific (assay ID: Hs02511055_s1, Hs04194492_g1, Hs01592477_m1, Hs02517015_s1, Hs01592482_m1, Hs02516855_sH, Hs02516990_s1, and Hs00870076_s1 for 8 UGTs; Hs02786624_g1 for GAPDH). The GAPDH gene was used as a reference gene. qRT-PCR was conducted on the Step-One Plus Real-Time PCR System (Applied Biosystems). The PCR reaction procedure was as follows: 95 °C for 20 s, followed by 45 cycles of 95 °C for 3 s, and 62 °C for 30 s. Expression data were normalized to GAPDH expression using the comparative Ct method to determine the relative mRNA expression of the eight UGTs.

#### 2.3.3. In Vivo Metabolism of IMM-H004 in Rat Brain after Intracerebroventricular Injection

The metabolism of IMM-H004 in the brain was investigated through the intracerebroventricular (icv) injection of IMM-H004 in rats, and the drug concentration in each brain region was analyzed. The rats were fixed on a benchmark stereotactic instrument (Leica Biosystems, Deer Park, IL, USA) under isoflurane anesthesia. Using a microinjector connected to a syringe pump, a 5 μL solution of IMM-H004 was dissolved in artificial cerebrospinal fluid (ACSF) and injected (3.8 μg/kg) into the lateral ventricle. The injection coordinates were as follows: medial/lateral = 1.5 mm; anterior/posterior = −0.8 mm; dorsal/ventral = −4 mm. Animals were sacrificed at 6, 15, 30, 45, and 60 min after drug administration. The brains were collected and dissected on ice into the following regions: the cerebellum, brainstem, hypothalamus, hippocampus, midbrain, striatum, and cortex. The subsequent procedure for processing tissue samples was the same as the procedure described in Section 2.3.1.

### 2.4. Transport Studies of IMM-H004 and IMM-H004G

#### 2.4.1. Studies in hCMEC/D3 Cells

##### Kinetic Studies

Uptake assays into hCMEC/D3 cells were conducted in 24-well plates. All incubations were carried out at 37 °C in triplicate unless otherwise noted. In time-dependent uptake studies, hCMEC/D3 cells were incubated with IMM-H004 (10 µmol/L) and IMM-H004G (50 µmol/L) dissolved in HBSS under gentle shaking for up to 30 min. To investigate the impact of incubation temperature on the uptake of IMM-H004 and IMM-H004G by hCMEC/D3 cells, incubation was performed at 4 or 37 °C for 10 min. To determine the kinetic parameters, the cells were incubated with IMM-H004 and IMM-H004G (2–400 µmol/L) for 10 min. The uptake data were fitted to the following equation using nonlinear least-square regression analysis in GraphPad Prism 9 (GraphPad, San Diego, CA, USA):

(1) $$V=\frac{V_{max}\times S}{K_{m}+S}+K_{ns}\times S$$

where *V* is the uptake velocity (nmol/mg protein/min), and *S* is the initial concentration (μmol/L). *V_max_*, *K_m_*, and *K_ns_* represent the maximum uptake velocity (nmol/mg protein/min), the Michaelis–Menten constant (μmol/L), and nonsaturable uptake clearance (μL/mg protein/min), respectively. The saturable uptake of both IMM-H004 and IMM-H004G was plotted with the Eadie–Hofstee method by subtracting passive uptake from the total uptake.

##### Identification of Transporters Using Inhibitors

The toxicity of all compounds, including inhibitors and substrates, toward hCMEC/D3 cells, was assessed using the 3-(4,5-Dimethylthiazol-2-yl)-2,5-diphenyltetrazolium bromide (MTT) reduction assay as described previously [11]. To assess the effects of pyrilamine-sensitive proton-coupled organic cation (H^+^/OC) antiporter, organic anion transporter (OAT), organic anion-transporting polypeptides (OATPs), organic cation transporter (OCT), and novel organic cation–carnitine transporter (OCTN) inhibitors on the uptake process, IMM-H004 or IMM-H004G (10 µmol/L) combined with the inhibitors were added to the medium after preincubating the cells with the inhibitors for 30 min. For evaluating the impact of the multidrug and toxin extrusion transporter (MATE), P-glycoprotein (P-gp), multidrug resistance protein (MRP), and breast cancer resistance protein (BCRP) on the uptake, IMM-H004 or IMM-H004G (10 µmol/L) was preincubated with the cells for 2 h before the addition of the inhibitors. After removing the supernatant, the cells were washed twice with prewarmed HBSS and incubated with fresh HBSS for 20 min. At the end of incubation, the supernatants were collected. The cells were washed with cold HBSS and lysed using RIPA buffer. The protein content of the lysed cells was determined using a bicinchoninic acid (BCA) assay kit (Beyotime Inc., Shanghai, China). The results were expressed as a percentage of the vehicle control. The substrates and inhibitors used herein are summarized in Table 1.

#### 2.4.2. Identification of Transporters Using Overexpressing Cells

CHO cells and MDCKⅡ cells were stably transfected with lentiviruses carrying human SCLO1A2 (encoding OATP1A2) and SCLO2B1 (encoding OATP2B1), respectively. Cell transfected with lentiviruses carrying empty vectors served as control. Lentiviral plasmid packages were obtained from OBiO Technology (Shanghai, China), and procedures followed the instructions of the virus manual. Stably transfected cells were routinely grown in a 100 mm dish in DMEM with 10% fetal bovine serum. Prior to the experiments, the seeding density of the overexpressing cells in 24-well plates was 4 × 10^5^ cells per well, and the medium was supplemented with 2 mM sodium butyrate for 24 h. The cultured cells were washed twice with prewarmed HBSS after removing the medium and then treated with 10 μmol/L IMM-H004G and 100 μmol/L rifampicin (or an equal amount of solvent dimethyl sulfoxide) for 10 min. After incubation, the cells were washed four times with cold HBSS. Follow-up sample processing procedures were as described in Section 2.3.1. E17βG (5 μmol/L) and rosuvastatin (10 μmol/L) were used as positive substrates in CHO-OATP1A2 and MDCKⅡ-OATP2B1 cells, respectively. The MDCKⅡ-MDR1 cells were previously established by our laboratory [11].

To further validate the effect of P-gp on the transport of IMM-H004G or IMM-H004, MDCKⅡ-MDR1 cells were seeded onto polycarbonate membrane transwell inserts in 24-well plates and used for transport studies after 5 days or when the effective transepithelial electric resistance (TEER) values exceeded 300 Ω·cm^2^. The monolayers were initially rinsed twice with HBSS. Subsequently, IMM-H004G or IMM-H004 (10 µmol/L) was added to either the apical or basolateral side of the monolayer. Aliquots of 50 µL were collected from the opposite compartment at various time points, covering a maximum duration of 120 min. Digoxin (5 μmol/L) was used as a positive substrate, while PSC833 (5 µmol/L) was used as an inhibitor in MDCKⅡ-MDR1 cells. The apparent permeability value (*P_app_*) was calculated using the following equation:

(2) $$P_{app}=\frac{dQ/dt}{{AC}_{0}}$$

where *dQ/dt* is the slope of the cumulative transport amount over time during the study period. *A* is the base area of the insert, and *C*_0_ is the initial addition concentration.

The efflux ratio (ER) was calculated as follows:

ER = *P_app_*_(*B*−*A*)_/*P_app_*_(*A*−*B*)_ (3)

#### 2.4.3. In Situ Brain Perfusion

The rat brain was perfused following a previously described method [31]. Briefly, the common carotid arteries on both sides were cannulated and connected to the perfusion system, while the external carotid artery was ligated. At the beginning of perfusion, the jugular veins on both sides were quickly incised. The perfusion procedure consisted of a preperfusion wash with saline for 1 min, followed by drug infusion (2 µmol/L IMM-H004 or IMM-H004G) for 2, 5, and 10 min, and concluded with a postperfusion wash (saline for 5 min) at a rate of 4.0 mL/min. To examine the impact of P-gp (PSC833, 5 µmol/L) and OATP (rifamycin SV, 100 µmol/L) on BBB transport, the perfusate containing the inhibitor was perfused for 1 min, and then the mixture of IMM-H004 or IMM-H004G (10 µmol/L) and the inhibitor was perfused for 10 min. Following the perfusion, the rats were decapitated, and their brains were homogenized (1:3, *w*/*v*) in chilled saline. The concentrations of IMM-H004 and IMM-H004G were then determined by LC-MS/MS.

The unidirectional transfer constant *K_in_* (mL/min/g) was calculated using the following equation:

(4) $$K_{in}=\frac{Q_{br}}{C_{pf}\times T}$$

where *Q_br_*/*C_pf_* is the apparent brain distribution volume, *Q_br_* is the measured amount of compound in brain tissue, *C_pf_* is the concentration of compound in the perfusate, and *T* is the perfusion time.

### 2.5. Pharmacokinetic Study in Rats

#### 2.5.1. Plasma Protein Binding

The rat plasma protein binding of IMM-H004 and IMM-H004G (0.5–10 μg/mL) was investigated using a rapid equilibrium dialysis (RED) assay. The RED plate inserts utilized in this assay contained a dialysis membrane with a molecular weight cut-off of 8 kDa (Thermo Fisher Scientific, Waltham, MA, USA). For the assay, aliquots of plasma (0.2 mL) and phosphate-buffered saline (0.35 mL) were added to the sample chamber and buffer chamber, respectively. The plate was then sealed and incubated at 37 °C, 200 rpm for 4 h. Following the dialysis period, the concentrations of IMM-H004 and IMM-H004G were determined using LC-MS/MS. The ratio of the drug concentration in the buffer to that in the plasma chamber was used to calculate the unbound fraction (fu) of the drugs.

#### 2.5.2. Pharmacokinetics

Rats were anesthetized with isoflurane. Microdialysis intracerebral guide cannulas and CMA 12 Elite Probe (PAES membrane with a MWCO of 20 kDa, 2 mm membrane length (CMA/Microdialysis AB, Stockholm, Sweden)) were implanted into the cerebral cortex at the coordinates relative to bregma: medial/lateral = 3.0 mm; anterior/posterior = 0.5 mm; dorsal/ventral = −3.0 mm. After a 24 h recovery period, the intracerebral probe was connected to the microdialysis cannula, and Ringer’s solution was perfused at a flow rate of 2.0 μL/min. Then, IMM-H004 citrate or IMM-H004G (10 mg/kg) was injected intravenously (iv). Dialysate samples were collected every 5 to 60 min for a duration of 7 h. The probe recovery in vitro was determined by dialyzing a standard mixture of IMM-H004 (50 ng/mL) and IMM-H004G (300 ng/mL) infused with Ringer’s solution.

Blood samples were collected from the orbital plexus into heparinized tubes and subsequently centrifuged to separate the plasma. The concentrations of IMM-H004 and IMM-H004G in both the dialysate and plasma samples were determined using LC-MS/MS analysis. Subsequently, the free concentrations in the brain and plasma were corrected with probe recovery and fu for further analysis, respectively. The pharmacokinetic parameters were calculated via noncompartmental analysis (NCA) using Phoenix WinNonlin 6.3 (Pharsight Corporation, Sunnyvale, CA, USA). The brain–plasma partition coefficient (Kp) was determined as the ratio of the area under the concentration–time curve for the brain (AUC_brain_) to that of the plasma (AUC_plasma_).

### 2.6. Determination of IMM-H004 and IMM-H004G Using LC-MS/MS

All samples were mixed with two volumes of ice-cold acetonitrile containing IS (propranolol, 0.1 μg/mL) and centrifugation at 18,800× *g* for 5 min. The supernatant containing IMM-H004 and IMM-H004G was analyzed using LC-MS/MS according to the previously reported methods [8,32]. In brief, the LC-MS/MS system consisted of an API 4000 mass spectrometer (AB SCIEX, Framingham, MA, USA) coupled with a Shimadzu UPLC LC-30A (Shimadzu Corporation, Kyoto, Japan). The analytical column used was a Zorbax Eclipse Plus C18 column (2.1 × 50 mm, 3.5 μm, Agilent, Santa Clara, CA, USA). The separation was performed at a flow rate of 0.3 mL/min. The mobile phase consisted of methanol and water both containing 0.5% formic acid. Quantification was carried out using the multiple reaction monitoring transition *m*/*z* 305.1→248.1 (IMM-H004), *m*/*z* 481.3→305.1 (IMM-H004G), and *m*/*z* 260.1→183.1 (propranolol, IS).

### 2.7. Statistical Analysis

Data were processed using Excel 2019 (Microsoft Inc., Washington, DC, USA) and are presented as mean ± SD. The statistical analysis of the data between the two groups was performed using an unpaired *t*-test. Statistical significance was considered as *p* < 0.05.

## 3. Results

### 3.1. Metabolism of IMM-H004 and IMM-H004G in Brain Cells and Brain Homogenate

**Figure 2:**
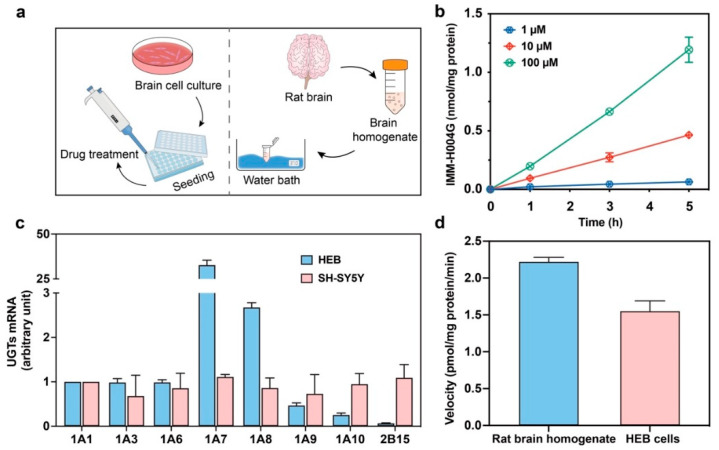
Metabolism of IMM-H004 and IMM-H004G in human brain cells and rat brain homogenate: (**a**) schematic diagram of the metabolism study in human brain cell lines and rat brain homogenate; (**b**) production of IMM-H004G when IMM-H004 (1–100 μmol/L) was incubated with HEB cells for up to 5 h (*n* = 2); (**c**) expression of UGTs mRNA in HEB and SH-SY5Y cells (*n* = 3); (**d**) in vitro generation rates of IMM-H004 to IMM-H004G in rat brain homogenate and HEB cells (*n* = 3).

Brain cells consist primarily of neurons, neuroglial cells, and cerebral endothelial cells. SH-SY5Y, HEB, and hCMEC/D3 are three commonly used human-derived cell lines, employed for the study of neurons, neuroglial cells, and cerebral microvascular endothelial cells, respectively. Thus, the metabolism of IMM-H004 and IMM-H004G in the brain and the species differences were evaluated using these three human brain cell lines and the rat brain homogenate (Figure 2a).

In the SH-SY5Y and hCMEC/D3 cell systems, IMM-H004G was not detected, while in the HEB system, the production of IMM-H004G depended on both time and concentration (Figure 2b). Conversely, when IMM-H004G was incubated with these cells for 24 h, no significant amounts of IMM-H004 were detected.

It was reported that human brain microvessels did not exhibit obvious expression of any UGT genes [33]. Since IMM-H004 can form its glucuronide conjugate in HEB cells, the expression of eight UGT genes involved in the glucuronidation of IMM-H004 was investigated in both HEB and SH-SY5Y cells to determine any intercellular difference. As shown in Figure 2c, all eight UGT isoforms were detected in HEB cells, with UGT1A7 being the predominant UGT gene expressed. A smaller amount of UGT1A8 transcript was detected (approximately 10-fold less), while the expression of other UGT genes was weak (0.3–3.5%). In comparison, SH-SY5Y cells exhibited detectable levels of all eight UGT genes but at much lower levels than HEB cells. In vitro studies using cultured human brain cell lines have revealed that the interconversion of IMM-H004 and IMM-H004G is primarily facilitated by UGTs, with a predominant transformation of IMM-H004 into IMM-H004G. This metabolic conversion predominantly occurs in neuroglial cells, where UGT1A7 is believed to play a crucial role in the glucuronidation of IMM-H004.

To investigate the potential species differences in the glucuronidation of IMM-H004 between rats and human brains, IMM-H004 (10 μmol/L) was incubated with the rat brain homogenate for 1 h. The formation of IMM-H004G was observed to be time-dependent. Interestingly, both the rat brain homogenate and HEB cells exhibited a similar metabolic rate in generating IMM-H004G, as depicted in Figure 2d. These results suggest that the rat brain may possess a comparable ability to humans in catalyzing the glucuronidation of IMM-H004.

Additionally, similar to the findings observed in human brain cell lines, incubating IMM-H004G (1 μmol/L) with the rat brain homogenate (2 mg/mL protein) for 4 h did not result in a reduction in its concentration (Supplementary Information, Figure S1). This provides additional evidence that the conversion of IMM-H004G back into IMM-H004 is difficult within the brain.

### 3.2. Metabolism of IMM-H004 after Intracerebroventricular Injection

**Figure 3:**
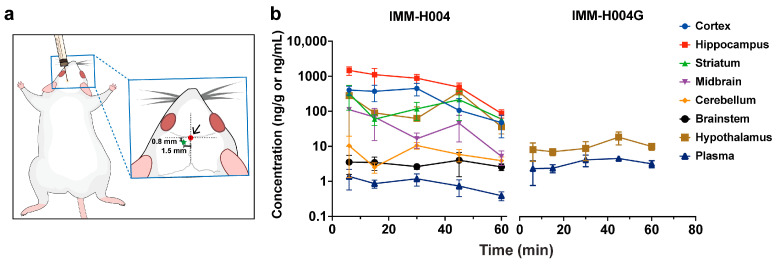
Metabolism of IMM-H004 after intracerebroventricular injection: (**a**) schematic diagram of intracerebroventricular injection. The red dot indicates the position of the bregma and the green pentagram represents the injection site; (**b**) concentration–time profiles of IMM-H004 and IMM-H004G in different regions of rat brain after intracerebroventricular injection of IMM-H004 at 3.8 μg/kg (*n* = 4).

It is established that icv injection can provide insights into the real-time metabolic state of drugs within the brain. To assess this, IMM-H004 was injected into the lateral ventricle of rats, and the concentrations of IMM-H004 and its glucuronide metabolite IMM-H004G in different brain regions were analyzed (Figure 3). The determination of dosage was based on the initial concentration of IMM-H004 in the brain following iv injection.

The results demonstrated that IMM-H004 was detected in all brain regions, including the cerebellum, brainstem, hypothalamus, hippocampus, midbrain, striatum, and cortex, within 6–60 min after icv injection. The highest concentration of IMM-H004 was found in the hippocampus near the injection site, followed by the cortex, striatum, and hypothalamus. Lower concentrations were observed in the midbrain, cerebellum, and brainstem. In contrast, IMM-H004G was only detected at lower concentrations in the hypothalamus. Additionally, trace amounts of IMM-H004 and IMM-H004G were also detected in the plasma. The hypothalamus-to-plasma AUC ratio of IMM-H004G was 3.13, suggesting that IMM-H004G in the hypothalamus originated from in situ metabolism. As IMM-H004G was exclusively detected in the hypothalamus after icv injection and at significantly lower levels than iv injection, these findings confirmed that the glucuronidation of IMM-H004 occurs in the brain but to a limited extent.

### 3.3. Uptake Kinetics in hCMEC/D3 Cells

Brain microvascular endothelial cells constitute the blood–brain barrier and express various transporters to protect the brain against exogenous substances. The hCMEC/D3 cell line, derived from isolated human brain capillary endothelial cells, is widely utilized as an in vitro model for studying the transport mechanisms of drugs across the BBB. This is attributed to its expression of 144 SLC transporters and 23 ABC efflux transporters [34]. To investigate the transport characteristics of IMM-H004 and IMM-H004G across the BBB, a study was conducted on the uptake kinetics of IMM-H004 and IMM-H004G in hCMEC/D3 cells.

**Figure 4:**
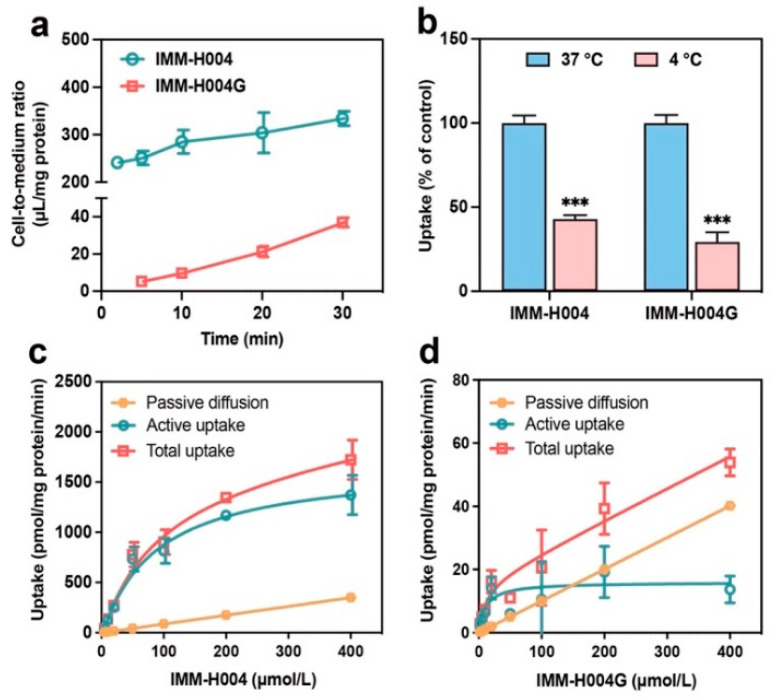
Uptake characteristics of IMM-H004 and IMM-H004G in hCMEC/D3 cells: (**a**) time-dependent uptake of IMM-H004 (10 μmol/L) and IMM-H004G (50 μmol/L); (**b**) the effect of temperature on the uptake of IMM-H004 and IMM-H004G (10 μmol/L, 10 min); (**c**,**d**) the concentration-dependent uptake of IMM-H004 and IMM-H004G (2–400 μmol/L, 10 min). *n* = 3. *** *p* < 0.001 vs. control group.

The uptake time profiles of IMM-H004 (10 μmol/L) and IMM-H004G (50 μmol/L) are illustrated in Figure 4a. It was found that the uptake of IMM-H004 was markedly higher than IMM-H004G. Moreover, both IMM-H004 and IMM-H004G exhibited time-dependent accumulation in hCMEC/D3 cells within 30 min. Therefore, a 10 min uptake period was selected for subsequent uptake kinetic and inhibition studies.

In general, energy consumption and active transport processes are minimal at temperatures between 0 and 4 °C. While the diffusion rate may diminish with a decreased temperature, the rate of mediated transport falls sharply. Therefore, comparing the uptake of substances at 4 °C and 37 °C can confirm the involvement of carrier-mediated transport processes in drug transport.

As shown in Figure 4b, the uptake of IMM-H004 and IMM-H004G showed a significant decrease at 4 °C, with respective reductions of 57.1% and 70.6%, compared to 37 °C (*p* < 0.01). These results suggest that the transport of these compounds is dependent on temperature and energy, implying that carrier-mediated processes contribute to their transport.

Furthermore, the concentration-dependent curves of IMM-H004 and IMM-H004G both exhibited an initial rapid increase, followed by a gentle, constant, and positive slope, indicating the occurrence of both saturable and unsaturated uptake (Figure 4c,d). The occurrence of saturable uptake of IMM-H004 and IMM-H004G further confirms the involvement of transporters in mediating the uptake of these compounds. The *K_m_* values for IMM-H004 and IMM-H004G were determined as 89.5 and 10.7 μmol/L, respectively, based on the calculated kinetic parameters. The *V_max_*/*K_m_* ratios for the transporter-mediated uptake of IMM-H004 and IMM-H004G were 18.8 and 1.50 μL/min/mg protein, respectively. The *K_ns_* values for the passive diffusion rate of IMM-H004 and IMM-H004G were 0.877 and 0.100 μL/min/mg protein, respectively. The *K_ns_* values for the passive diffusion rate of IMM-H004 were 8.77 times that of IMM-H004G, indicating the greater membrane permeability of IMM-H004.

### 3.4. Identification of the Transporters in hCMEC/D3 Cells by Inhibitors

To investigate the transport mechanisms of IMM-H004 and IMM-H004G in more detail, we performed inhibition assays on nine transporters that exhibit the highest expression levels in the BBB and are involved in drug transport. All the positive substrates and inhibitors used in this study were tested at nontoxic concentrations, as confirmed by the MTT assay with a cell survival rate greater than 90%. DPH, metformin, TEA, methotrexate, E3S, digoxin, and mitoxantrone were employed as substrates for pyrilamine-sensitive H^+^/OC antiporter, OCT and MATE, OCTN, OAT and MRP, OATP, P-gp, and BCRP, respectively.

**Figure 5:**
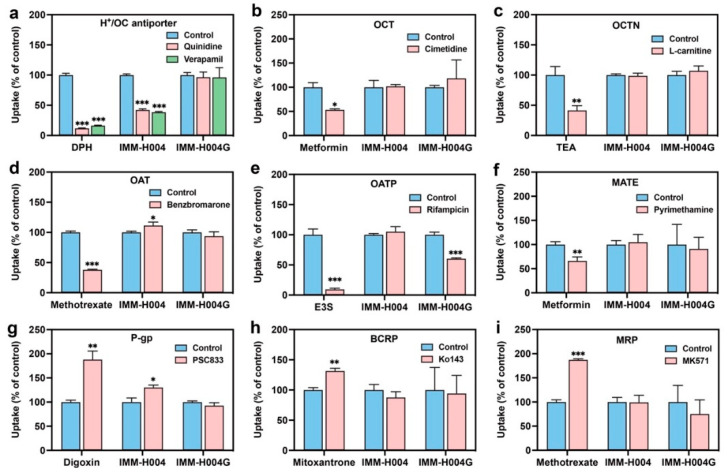
Effect of transporter inhibitors on the accumulation of IMM-H004 and IMM-H004G in hCMEC/D3 cells: (**a**) DPH (diphenhydramine, 1 μmol/L) is the substrate, and quinidine and verapamil (100 μmol/L) are the inhibitors of H^+^/OC antiporter; (**b**) metformin (10 μmol/L) and cimetidine (100 μmol/L) are the substrate and inhibitor of OCT, respectively; (**c**) TEA (tetraethylammonium, 20 μmol/L) and L-carnitine (5 μmol/L) are the substrate and inhibitor of OCTN, respectively; (**d**) methotrexate (10 μmol/L) and benzbromarone (10 μmol/L) are the substrate and inhibitor of OAT, respectively; (**e**) E3S (estrone 3-sulfate, 5 μmol/L) and rifamycin (100 μmol/L) are the substrate and inhibitor of OATP, respectively; (**f**) metformin (10 μmol/L) and pyrimethamine (1 μmol/L) are the substrate and inhibitor of MATE, respectively; (**g**) digoxin (5 μmol/L) and PSC833 (5 μmol/L) are the substrate and inhibitor of P-gp, respectively; (**h**) mitoxantrone (5 μmol/L) and Ko143 (5 μmol/L) are the substrate and inhibitor of BCRP, respectively; (**i**) methotrexate (10 μmol/L) and MK571 (10 μmol/L) are the substrate and inhibitor of BCRP, respectively. *n* = 3. *** *p* < 0.001, ** *p* < 0.01, * *p* < 0.05 vs. control group.

As shown in Figure 5, the uptake or efflux of positive substrates in hCMEC/D3 cells decreased by 30–90% in the presence of corresponding inhibitors, confirming the reliability of the experiment system. Notably, quinidine and verapamil, inhibitors of the pyrilamine-sensitive H^+^/OC antiporter, reduced the uptake of IMM-H004 by 57.7% and 61.5%, respectively, indicating the involvement of the pyrilamine-sensitive H^+^/OC antiporter in the uptake transport of IMM-H004. However, the uptake of IMM-H004G was not affected by these inhibitors. Interestingly, the OATP inhibitor rifamycin resulted in a 39.6% reduction in the uptake of IMM-H004G. This suggests that IMM-H004G may be a substrate for the OATP uptake transporter. Moreover, the P-gp inhibitor PSC833 increased the uptake of IMM-H004 to 1.3 times that of the control, without affecting the uptake of IMM-H004G. This indicates that P-gp is selectively involved in the efflux of IMM-H004. No significant impact on the uptake of IMM-H004 and IMM-H004G was observed with inhibitors targeting other transporters, including OAT, OCT, OCTN, BCRP, and MRP. This suggests that these transporters are unlikely to be associated with the transport of IMM-H004 and IMM-H004G.

### 3.5. Transport in Overexpressing Cells

**Figure 6:**
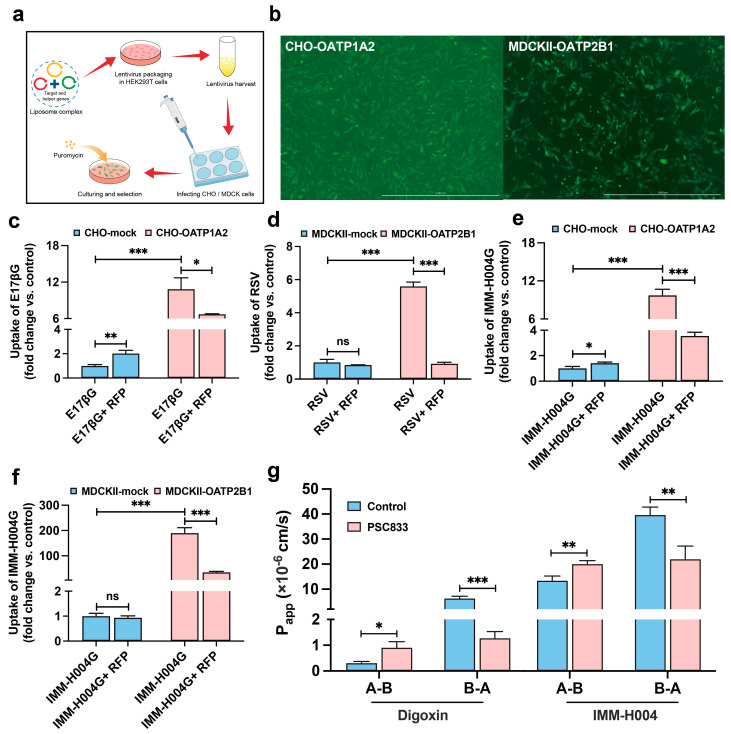
Transmembrane process of IMM-H004 and IMM-H004G mediated by P-gp and OATPs: (**a**) schematic diagram of the construction of transporter-overexpressing cells; (**b**) the photographs of green fluorescent protein (GFP) of CHO-OATP1A2 and MDCKⅡ-OATP2B1 cells, respectively, scale bar = 1000 μm; (**c**) E17βG (estradiol-17β-glucuronide, 5 μmol/L) was used as a positive group in CHO cells; (**d**) RSV (rosuvastatin, 10 μmol/L) was used as a positive group in MDCKⅡ cells; (**e**,**f**) the uptake of IMM-H004G (10 μmol/L) in OATP-overexpressing and mock cells; RFP (rifampicin, 100 μmol/L) served as a pan-OATP inhibitor; (**g**) the *P_app_* values of IMM-H004 (10 μmol/L) in monolayers of MDCKⅡ-MDR1 cells. *n* = 3. *** *p* < 0.001, ** *p* < 0.01, * *p* < 0.05 vs. control group. ns, not significant.

To further explore the potential role of OATPs in facilitating the entry of IMM-H004G into the brain, we successfully constructed CHO-OATP1A2 and MDCKⅡ-OATP2B1 cells overexpressing these transporters using a lentiviral vector system (Figure 6a,b). Overexpression was validated by the increased uptake of positive substrates, showing a 10.8-fold and 5.6-fold increase compared to the control cells (Figure 6c,d). In OATP1A2- and OATP2B1-overexpressing cells, the IMM-H004G uptake was markedly higher, with 9.7-fold and 190-fold increases compared to the control cells, respectively (Figure 6e,f), suggesting that IMM-H004G is a substrate for OATP and exhibits stronger selectivity toward OATP2B1.

The MDCKⅡ-MDR1 cell model is commonly used for identifying P-gp substrates. In the presence of PSC833, a P-gp inhibitor, the ER of digoxin, a known P-gp substrate, decreased from 21.14 to 1.41, confirming the reliable P-gp transport activity of the system (Figure 6g). The ER of IMM-H004 was 2.96, exceeding the threshold ER of 2, and it decreased to 1.10 in the presence of the P-gp inhibitor, indicating that IMM-H004 has the potential to be a P-gp substrate.

### 3.6. In Situ Brain Perfusion in Rats

**Figure 7:**
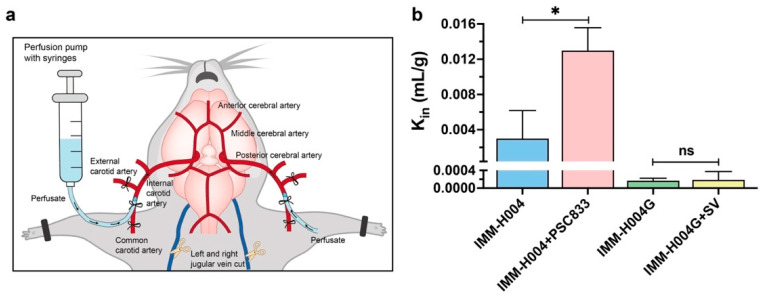
In situ brain perfusion of IMM-H004 and IMM-H004G in rats: (**a**) schematic diagram of in situ brain perfusion; (**b**) effect of P-gp and OATP inhibitors on the uptake characteristics of IMM-H004 and IMM-H004G using the in situ brain perfusion method. The concentration of IMM-H004 and IMM-H004G was 10 μmol/L. The concentrations of P-gp inhibitor PSC833 and the OATP inhibitor rifamycin SV were 5 μmol/L and 100 μmol/L, respectively. *n* = 3. * *p* < 0.05 vs. control group. ns, not significant.

In situ brain perfusion studies can comprehensively evaluate the influence of the physicochemical properties and transporter effects of IMM-H004 and IMM-H004G on their entry into the brain (Figure 7a). Both IMM-H004 and IMM-H004G exhibited time-dependent BBB crossing within 10 min (Supplementary Information Figure S2). As displayed in Figure 7b, the *K_in_* value of IMM-H004 was 18 times higher than that of IMM-H004G, indicating a faster rate of entry into the brain for IMM-H004, based on the drug’s physicochemical properties and transporter effects. Furthermore, the P-gp inhibitor PSC833 led to an increase in the *K_in_* value of IMM-H004 by 4.3 times compared to the control, suggesting that P-gp extremely limited the penetration of IMM-H004 into the brain. On the other hand, when an OATP inhibitor was added to the perfusion solution, there was no significant change in the *K_in_* value of IMM-H004G, demonstrating that the facilitating effect of OATP on the brain uptake of IMM-H004G in rats may be limited.

### 3.7. Pharmacokinetics in Rats

**Figure 8:**
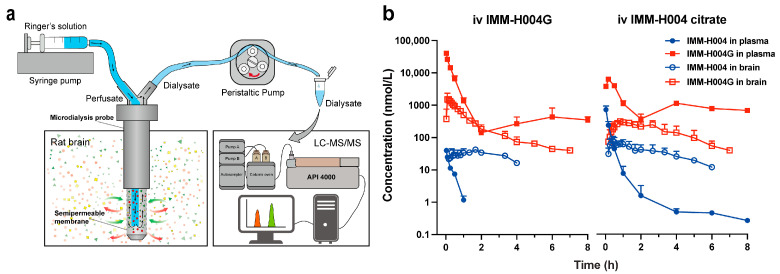
Pharmacokinetics of IMM-H004 and IMM-H004G in rats: (**a**) schematic diagram of the cerebral microdialysis technique; (**b**) free concentration–time profiles of IMM-H004 and IMM-H004G following intravenous injection of IMM-H004G and IMM-H004 citrate at a dose of 10 mg/kg (*n* = 3).

To gain a deeper understanding of the origin and brain entry mechanism of IMM-H004G, separate intravenous injections of IMM-H004 and IMM-H004G were administered to rats. The microdialysis technique was employed to assess the levels of the drugs in the brain, which measures the free drug concentration (Figure 8a). The fu values of IMM-H004 and IMM-H004G in rat plasma (0.5–10 μg/mL) were determined as 0.025 ± 0.002 and 0.315 ± 0.008, respectively (Supplementary Information, Figure S3). Consequently, the total plasma concentration was converted to the free plasma concentration using the fu value, and the pharmacokinetic parameters were calculated based on the free plasma concentration.

As presented in Figure 8b, following the iv injection of IMM-H004G, both IMM-H004G and a trace amount of IMM-H004 were detected in the plasma and brain. The plasma concentrations of IMM-H004G and IMM-H004 at 2 min were 40,215 nmol/L and 39.8 nmol/L, respectively. The peak brain concentrations were 1712 nmol/L and 35.1 nmol/L, respectively. The AUC_brain_ values were 1923 and 93.8 nmol/L·h, respectively. The higher concentration of IMM-H004G observed in the brain after intravenous injection indicates that IMM-H004G can penetrate the BBB and be distributed in the brain without depending on the in situ metabolism of IMM-H004.

After the iv injection of IMM-H004 citrate, The AUC_plasma_ values for IMM-H004G and IMM-H004 were determined as 16,418 and 141 nmol/L·h, respectively. The AUC_brain_ values were 1053 and 196 nmol/L·h, respectively. The Kp (AUC_brain_/AUC_plasma_) values for IMM-H004G and IMM-H004 were 0.06 and 1.39, respectively. Notably, the AUC_plasma_ and AUC_brain_ for IMM-H004G were 116 times and 5.4 times higher than those of IMM-H004, respectively.

## 4. Discussion

Glucuronidation, catalyzed by UDP glucuronosyltransferases (UGTs), is the most important phase II metabolic pathway [35]. Although glucuronidation has traditionally been viewed as a drug detoxification mechanism, there is a growing recognition that certain glucuronidation products possess pharmacological effects, such as anti-inflammatory, antioxidant, and antitumor activities, serving as the basis for the efficacy of drugs [36,37,38,39,40,41,42]. Particularly, certain active glucuronide conjugates can also exert effects on the central nervous system (CNS). For instance, morphine-6-glucuronide (M6G) acts as a potent agonist of opioid receptors and is involved in the analgesic process of morphine [43]. Quercetin-3-O-β-D-glucuronide (Q3GA), a metabolite of orally administered quercetin in rats, can penetrate the blood–brain barrier, improve oxidative stress in the brain, and exert neuroprotective effects [44].

UGTs have been detected in the brains of humans and various animal species, including mice, rats, and macaques [45]. Some studies suggested that several drugs, such as morphine, resveratrol, and efavirenz, can generate glucuronide conjugates in the brain microsomes, tissue homogenates, or cells [46,47,48]. Although their activity in the brain is lower than in the liver or other extra-hepatic organs, and UGTs have a minor impact on the overall glucuronidation of xenobiotics, they surely play a crucial role in maintaining the steady state of endogenous compounds [49,50]. It is speculated that UGTs can also have a major effect on the local efficacy of substances that enter the brain. Therefore, the primary purpose of this study was to explore the possibility of the in situ metabolism of IMM-H004 into IMM-H004G after entering brain tissue.

The location of UGT expression in brain tissue is controversial [3,4,5,32,49]. According to de Leon [51], UGTs are present in relatively low levels within brain tissue but appear to be more concentrated at brain capillaries, playing a role in the BBB. Ouzzine et al. [50] also reported that UGTs are primarily expressed in endothelial cells and astrocytes. Even in brain microvessel endothelial cells, there has been evidence of lamotrigine glucuronidation activity [52]. However, Shawahna et al. [33] did not observe any expression of UGT genes in human brain microvessels. The current consensus suggests that UGT activity is present in both neuronal and glial cells, with significantly higher activity in glial cells than in neurons. Our in vitro studies indicate the detection of UGT1A7 expression in human glial cells, with minimal expression observed in human neuronal cells. Furthermore, only the glial cells demonstrated the ability to glucuronidate IMM-H004. Similarly, UGT1A7 mRNA expression has been observed in rat astrocytes [53], and glucuronidation activity toward IMM-H004 has been detected in the rat brain, suggesting the consistent glucuronidation of IMM-H004 in both rat and human brains. To further uncover the glucuronidation activity in different brain regions, Asai et al. [54] compared the glucuronidation activity of *β*-estradiol, a UGT1A substrate, in microsomal proteins from various brain regions in rats. They observed that the hippocampus exhibited the highest activity, which was approximately twice that of the cerebrum. Consistently, following the icv injection of IMM-H004 in rats, IMM-H004G was only detected in the hippocampus, indicating the highest activity in the hippocampus and thus pointing to the limited glucuronidation of IMM-H004 in the brain.

Due to the tight junctions in the endothelial cells of the BBB, drugs in the vascular system cannot enter through the gaps between microvascular endothelial cells. They can only pass through the cell membrane. After undergoing glucuronidation, drugs have increased molecular weight and polarity, which theoretically poses a challenge for their entry into the brain. Based on the physicochemical properties, IMM-H004G is not favored for passive diffusion across the BBB; thus, it may require a transport system to achieve high concentrations in the brain.

Transporters presented on the BBB can be divided into two categories: ATP-binding cassette (ABC) superfamily transporters and solute carrier (SLC) superfamily transporters. ABC transporters, including P-gp, BCRP, and MRPs, mediate drug efflux transport, while SLC transporters, serving as bidirectional or uptake transporters, facilitate drug entry into the brain. The top four SLC transporters include glucose transporter 1 (GLUT1), L-type amino acid transporter 1 (LAT1), monocarboxylate transporters (MCTs), and nucleoside transporters (ENTs), which are primarily involved in the transportation of endogenous substances in the brain [55,56]. Following these, OATP2B1, OATP1A2, OCTs, and OCTNs participate in the brain transport of drugs such as nefazodone, zolmitriptan, lamotrigine, and memantine. There is limited knowledge about the brain transport of glucuronide conjugates. According to the literature, M6G is reported to be a substrate for GLUT-1 and Oatp2, while 17β-estradiol and its glucuronide metabolite, as well as edaravone glucuronide, are substrates for Oatp2 and MRP4, respectively [57,58,59].

**Figure 9:**
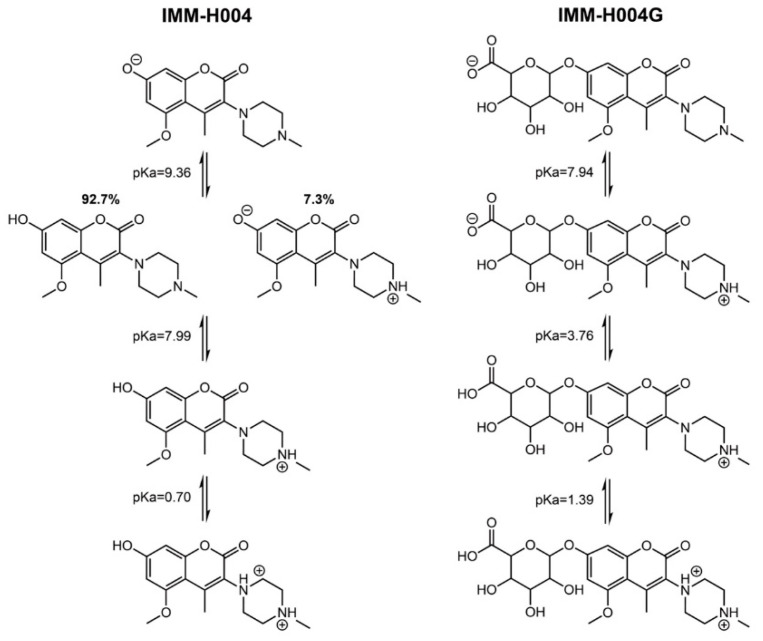
Software prediction of pKa values for IMM-H004 and IMM-H004G (2-column fitting image; color is not needed).

Both IMM-H004 and IMM-H004G are amphoteric molecules, each containing a piperazine ring as the basic group and featuring a phenolic hydroxyl and carboxyl group as the acidic groups, respectively. The analysis using ADMET Predictor 8.5 software (Simulations Plus, Inc., Lancaster, CA, USA) showed that, under physiological conditions (pH = 7.4), IMM-H004 and IMM-H004G may mainly exist in cationic form and zwitterionic form, respectively (Figure 9). Correspondingly, inhibitor assays conducted in hCMEC/D3 cells revealed that the uptake of IMM-H004 and IMM-H004G is primarily mediated by the H^+^/OC antiporter and OATPs, respectively. Other cationic transporters, such as OCT and OCTN, do not play a role in the transport of either compound. Additionally, P-gp is potentially involved in the efflux of IMM-H004. Although the glucose transporter GLUT has been reported to be involved in the transport of morphine-6-glucuronide [57], ourunpublished experiments using GLUT1-silenced hCMEC/D3 cells did not yield positive results.

Overexpressing cells are an effective tool for further validating the function and involvement of transporters. Regrettably, the sequence and encoding gene for the pyrilamine-sensitive H^+^/OC antiporter protein have not been determined yet [60]. Consequently, it is currently impossible to construct an overexpression cell line to confirm its involvement in the uptake of IMM-H004. However, we validated the efflux effect of P-gp protein on IMM-H004 using the MDCKⅡ-MDR1 cell transwell model.

OATP1A2 and OATP2B1 are considered to be the most abundant OATPs at the BBB. OATP1A2 is predominantly localized on the apical side of the brain capillary endothelial cells, while OATP2B1 is primarily distributed on the basolateral sides [61]. Although both OATP1A2 and OATP2B1 have been implicated in the transport of neuroactive steroids and various exogenous compounds including statins, antidiabetics, antihistamines, and central active drugs like triptans [61], there are still subtle differences between them. OATP1A2 exhibits broad substrate specificity for the BBB, while OATP2B1 has a relatively narrower substrate specificity [62].

Consistent with the findings from the OATP inhibitor assay in hCMEC/D3 cells, the uptake of IMM-H004G was significantly higher in cells overexpressing OATP1A2 and OATP2B1 compared to the control cells. Particularly noteworthy was the striking 190-fold increase in uptake observed in MDCKⅡ-OATP2B1 cells compared to the control cells. Furthermore, we explored the involvement of other OATPs by using MDCKⅡ cells overexpressing OATP1B1 and OATP1B3. Although the uptake of IMM-H004G by both cell lines was notably higher than that of the control cells, it did not exceed a tenfold increase (Supplementary Information, Figure S4). Taken together, these results indicate that IMM-H004G may have a significantly higher selectivity for OATP2B1 than other OATP subtypes.

Drug permeability across the BBB is primarily governed by passive diffusion and carrier-mediated transport. Compared to cellular models, which are valuable tools for examining the impact of individual factors, animal models can offer a more comprehensive understanding of drug permeation. In situ brain perfusion is widely regarded as the gold standard method for measuring BBB permeability [63]. Therefore, we conducted in situ rat brain perfusion studies. Consistent with the uptake kinetic assay results using hCMEC/D3 cells (*K_ns_* values), the *K_in_* value of IMM-H004 was markedly higher than that of IMM-H004G, highlighting the higher brain entry rate of IMM-H004. The addition of the P-gp inhibitor PSC833 noticeably increased the *K_in_* value of IMM-H004, underscoring the significant role of P-gp-mediated efflux in limiting the overall accumulation and extent of IMM-H004 penetration into the brain. Conversely, IMM-H004G, despite its slower entry rate, achieved a greater extent of brain exposure, which is attributed to the lesser effect of efflux transporters such as P-gp on its BBB permeability. However, the OATP inhibitor showed no substantial effect on the entry process of IMM-H004G into the brain.

In contrast to what is known for humans, very little is known about the substrate specificity and affinity of rodent orthologues of OATP2B1. Some researchers argue that species differences should be taken into consideration when studying OATP2B1 [64]. For example, Hussner et al. [65] reported that there are species differences in the recognition of OATP2B1 transporter for steroid sulfate conjugates between rats and humans. Since IMM-H004G exhibits significantly higher selectivity for OATP2B1 than for other OATPs, we speculate that species differences in OATP2B1 may be a key factor contributing to the negative result of the OATP inhibition assay in the rat brain perfusion. Humanized animal models may provide a feasible solution for addressing potential species differences.

Based on tissue cells containing β-glucuronidase activity that can hydrolyze glucuronide conjugates to produce aglycones, Terao et al. [66] proposed that glucuronide conjugates of quercetin serve as prodrugs in the vascular system, and their pharmacological effects are exerted through hydrolysis to release the parent drug. In the case of IMM-H004G, a glucuronide conjugate that exhibits higher brain exposure than its parent form with similar activity both in vitro and in vivo, the interconversion of IMM-H004 and IMM-H004G in the brain was investigated. Our findings demonstrate that IMM-H004G cannot be hydrolyzed back into IMM-H004 in either rat brain tissue homogenates or human brain cells, suggesting that IMM-H004G exerts pharmacological effects on the brain on its own.

## 5. Conclusions

The formation of IMM-H004G in the brain was confirmed in the present study. More importantly, although IMM-H004G had a slower entry rate, it achieved a greater extent of exposure in the brain. This is because its BBB permeability is less affected by efflux transporters such as P-gp, and it benefits from OATP uptake, enabling it to exert its independent pharmacological effects. Overall, these findings deepen our understanding of the metabolism and transport mechanisms underlying CNS drugs and provide valuable insights for the development of CNS drugs that undergo glucuronidation.

## Figures and Tables

**Table 1:** Concentrations of the substrates and inhibitors for transporter identification assays in hCMEC/D3 cells.

Transporter	Substrate	Concentration (μmol/L)	Inhibitor	Concentration (μmol/L)	Reference
Pyrilamine-sensitive H^+^/OC antiporter	Diphenhydramine	1	Quinidine, Verapamil	100	[12,13,14]
OAT	Methotrexate	10	Benzbromarone	10	[15,16]
OCT	Metformin	10	Cimetidine	100	[17,18]
OCTN	Tetraethylammonium	20	L-Carnitine	5	[19,20,21]
OATP	Estrone 3-sulfate	5	Rifampicin	100	[22,23]
MATE	Metformin	10	Pyrimethamine	1	[24]
P-gp	Digoxin	5	PSC833	5	[25,26]
BCRP	Mitoxantrone	5	Ko143	5	[27,28]
MRP	Methotrexate	10	MK571	10	[29,30]

## Data Availability

Data will be available upon rational request.

## Supplementary Materials

The following supporting information can be downloaded at: https://www.mdpi.com/article/10.3390/pharmaceutics16030330/s1, Figure S1: Incubation of IMM-H004G (1 μmol/L) in rat brain homogenate (2 mg/mL protein) for up to 4 h (*n* = 3). Figure S2: In situ brain perfusion of IMM-H004 and IMM-H004G (2 μmol/L) in rats for up to 10 min (*n* = 3). Figure S3: Rat plasma protein binding and free fractions (fu values) of IMM-H004 and IMM-H004G (0.5–10 μg/mL, *n* = 3). Figure S4: Uptake of IMM-H004G in MDCKⅡ-OATP1B1 and MDCKⅡ-OATP1B3 cells.
