# Supplementary material for: The Active Glucuronide Metabolite of the Brain Protectant IMM-H004 with Poor Blood–Brain Barrier Permeability Demonstrates a High Partition in the Rat Brain via Multiple Mechanisms
Source: Pharmaceutics. 2024 Feb 27;16(3):330. doi: 10.3390/pharmaceutics16030330 (PMC10975012; doi:10.3390/pharmaceutics16030330)
Supplement: Supplementary file 1 [file pharmaceutics-16-00330-s001.zip › pharmaceutics-2855439-supplementary.pdf]

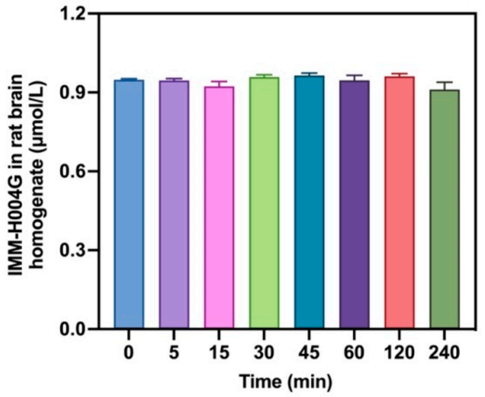

Figure S1 Incubation of IMM-H004G (1 μmol/L) in rat brain homogenate (2 mg/mL protein) for up to 4 h (*n*=3).

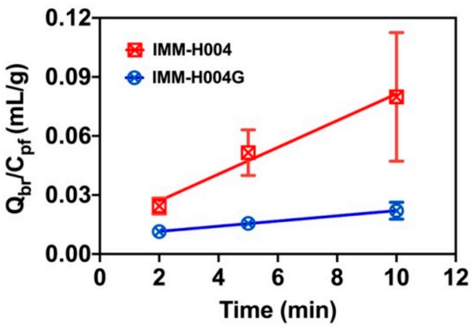

Figure S2 In situ brain perfusion of IMM-H004 and IMM-H004G (2 μmol/L) in rats for up to 10 min (*n*=3).

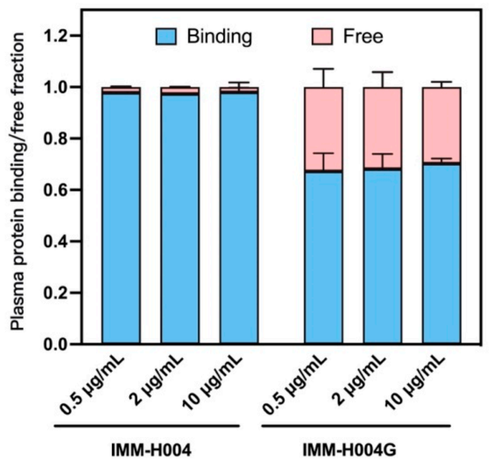

Figure S3 Rat plasma protein binding and free fractions (fu values) of IMM-H004 and IMM-H004G (0.5-10 μg/mL, *n*=3).

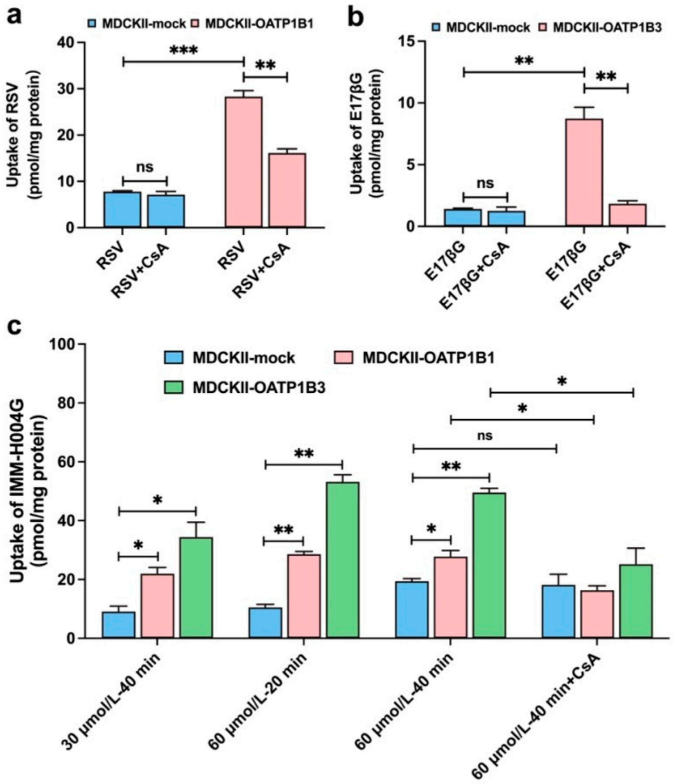

Figure S4 Uptake of IMM-H004G in MDCKII-OATP1B1 and MDCKII-OATP1B3 cells. (a) RSV (rosuvastatin, 30 μmol/L) is used as a positive group in MDCKII-OATP1B1 cells. (b) E17βG (estradiol-17β-glucuronide, 5 μmol/L) is used as a positive group in MDCKII-OATP1B3 cells. (c) IMM-H004G (30, 60 μmol/L) was incubated with MDCKII-OATP1B1 and MDCKII-OATP1B3 cells for 20 or 40 min. The OATP pan-inhibitor CsA (cyclosporin A) is 10 μmol/L. n=3 for positive substrates, and n=2 for IMM-H004G. ***p < 0.001, **p < 0.01, *p < 0.05 vs. control group. ns, not significant.
